# You Really Affect Me: The Role of Social Influence in the Relationship between Procedural Justice and Turnover Intention

**DOI:** 10.3390/ijerph19095162

**Published:** 2022-04-24

**Authors:** Hassane Gharbi, Nadir Aliane, Khaled A. Al Falah, Abu Elnasr E. Sobaih

**Affiliations:** 1 Management Department, College of Business Administration, King Faisal University, Al-Ahsaa 31982, Saudi Arabia; hgharbi@kfu.edu.sa (H.G.); nhaliane@kfu.edu.sa (N.A.); kalfalah@kfu.edu.sa (K.A.A.F.); 2 School of Business, University of Sfax, Sfax 3018, Tunisia; 3 Faculty of Tourism and Hotel Management, Helwan University, Cairo 12612, Egypt

**Keywords:** procedural justice, social influence, turnover intention, banking industry

## Abstract

This research aims to test the impact of procedural justice on employees’ turnover intention via their intention to stay or give up their positions by putting social influence in the spotlight as a mediating variable. Although the topic dealing with the relationship linking organizational justice to turnover intention has taken some wrinkles, there has been no published research, to the best of researchers’ knowledge, that integrates social influence as a mediating variable between the aforementioned relationships. A questionnaire survey was administered to 558 employees working in a renowned banking institution located in the capital city of Tunis, Tunisia. Structural equation modeling (SEM) results using AMOS software, IBM, version 23, showed that social influence partially mediated the relationship between procedural justice and turnover intention. More specifically, procedural justice has a significant negative effect on turnover intention (β = −0.30, *p* < 0.01) and a significant positive effect on social influence (β = +0.54, *p* < 0.01), which will have a significant positive effect on turnover intention (β = +0.91, *p* < 0.01). The results confirm that procedural justice is necessary for any organization; however, it is not enough to eliminate the turnover intention, especially with the presence of social influence. Social influence alters the judgments of those caught in its nets. This intangible aspect and power is even more enigmatic and harmful, which can lead to a change in cognitive references and behaviors. Social influence heavily affects the spontaneity of individuals and they became subject to dominant forces, which has to be properly controlled by management.

## 1. Introduction

Like a specter that haunts top management, turnover, in a context of labor deficiency [1,2], has become an important issue over the last few decades [3,4,5,6]. Employees are increasingly characterized by a desire to leave their job [7,8]. Moreover, numerous studies [2,9,10,11] show that turnover is an additional cost for the organization. Indeed, when an employee leaves the organization, management can do nothing, albeit spending money to recruit and train a new employee [12]. Additionally, it can lead to negative job outcomes, e.g., unethical behavior, social loafing behaviour and low job performance [13,14,15]. Many researchers (e.g., [16,17,18,19,20,21,22]) showed, from the results of their research, that procedural justice is one of the antecedents of intention to leave.

In this research, we will draw on Folger’s theory of Cognitive References (1986) [23], that the more the procedures applied are respected and perceived as fair, the lower the intention to turnover. Nevertheless, what about when procedural justice is honored while the turnover intention, supposedly weakening, strangely continues to increase? We assume here the existence of a mysterious power, even more effective as it is invisible, that Bellenger [24] calls social influence. Indeed, since one of the objectives of the individual is to escape ostracism, in the sense of Williams [25]. Therefore, s/he wants to be accepted by the group and to be positively appreciated by the members who constitute it. His gregarious instinct and the need to belong [26], hence, predispose her/him to subject himself/herself to the influence of the organizational climate. This influence can reach a deep degree by being at the level of the beliefs of the individual. The latter becomes an instrument of the will of others. As a result, social influence, driven by shared values and representations, guides individual and collective behavior. Therefore, this research aims to test the impact of procedural justice on employees’ turnover intention via their intention to stay or to give up their positions by introducing social influence as a mediating variable.

## 2. Definitions of Constructs

By definition, turnover refers to a conscious and deliberate desire to leave one’s organization. The literature [27,28,29] shows that turnover intention is difficult to detect [3] and is much more dangerous than turnover itself [30]. Employees intending to leave their organization may not declare it verbally while displaying various potential behavioral indicators of this intention. One of these indicators is counterproductive work behavior, thus reducing the performance of the organization [31] not to mention the associated deviant, unethical or social loafing behaviors [13,14,32], sabotage, withdrawal and harassment [33,34] are examples. In the same train of thought, Fishbein and Ajzen [35] announce, “the best predictor of an individual’s behavior will be a measure of his intention to adopt this behavior” (p. 369).

Turnover can be inherent to several factors [36,37]. Indeed, Amponsah-Tawiah et al.’s [38] research results postulate that it is the positive alliance of dissatisfied employees and a stressful work environment that leads to retirement intentions. Wen et al. [39] argue that turnover is strongly influenced by the way employees perceive their organizational climate and manage their work problems. In a similar vein, Effendi et al. [40] find that perceptions can be significantly affected by how employees interpret their work environment as positive, which helps them cope with work challenges and dampen quitting intentions. Hirst et al. [41] claim that the employee–supervisor relationship has a significant impact on employee turnover. Kim and Vandenberghe [42] instead focus on the ethical climate of the company, which, according to them, conditions the intention of the employee to stay or leave the company. Al Muharraq et al. [43] believe that the intention to leave is dependent on the workplace bullying experienced by employees in their organizations. Although the list of factors cannot be exhaustive in this article, we will focus on organizational justice, and more precisely, on procedural justice as a variable dependent on turnover. Moreover, in the literature, procedural justice appears as an antecedent to turnover [16,44,45].

Organizational justice has been slow to emerge. We had to wait until the end of the 1980s and the beginning of the 1990s for the structuring of a stream of research dealing with issues that directly concern the organization. This recent character of research in the field of organizational justice is at the origin of the debate, which drags on the dimensionality of this paradigm. Over the years, forms of organizational justice have arisen to depict the process of justice that binds an employee to his or her organization. The three fields of study of the latter are distributive, procedural and interactional justice [46,47,48]. Distributive justice refers to the basic principles when it comes to fairness: “Did I get what I deserved?” [49,50]. Procedural justice refers to the fairness of the procedures used to make and implement appropriate decisions and policies: “Do the rules and regulations treat me fairly?” [51]. Interactional justice is concerned with the quality of treatment received from decision-makers and the extent to which decision-making procedures are correctly represented: “Did they treat me fairly?” [52,53].

At the end of the 19th century, influence became one of the mascot domains of the social sciences. The power of influence, unlike that of persuasion, targets the spontaneity of the parties in relation. In this regard, Cialdini [54] concludes that influence lies in the power to “get people to say yes without thinking”. According to the *Dictionary of Human Sciences*, “influencia” in Medieval Latin, designated the occult power attributed to the stars to modify the destiny of men. Since then, the word has come down to earth. It has been humanized to designate the ability of everyone to change the ideas or actions of others, often without their knowledge. In this sense, influencing is tantamount to exerting an action on others. Since it is almost obvious that anything can influence us (a situation, a group, a person, a context, a policy, a tone, a set of terms, a certain way of behaving, a manner, a charisma, etc.).

Bellenger [24] shows that the degree of suggestion ability specific to everyone plays a preponderant role in the self-control of the process of influence. Therefore, he assumes that, inevitably, faced with the same source of influence, some individuals are more easily influenced than others are. In 1952, the “Ash effect”, alone, demonstrated that the group, through its influence, was able to change the opinion of an individual a priori certain of the correctness of his response to a prosaic problem, as long as he imitates the others, despite his conviction of the falsity of their answer. In his work entitled *Laws of Imitation*, Tarde [55] depicts the inexplicable determination of human beings to submit voluntarily to social influence in order to escape social disapproval.

## 3. Review of Literature and Formulation of Research Hypotheses

**Figure 1:**
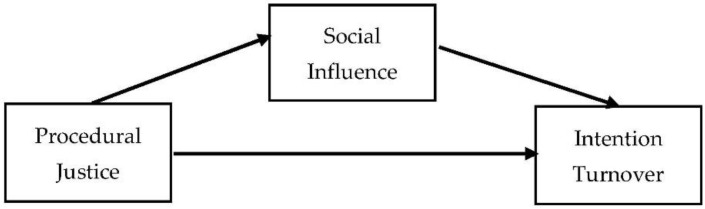
The research conceptual framework.

The theory of social exchange [56,57,58] starts from the postulate of reciprocity [59], stipulating that employees who perceive justice of any kind in their organization feel compelled to continue to be part of it. Conversely, an intention to leave emerges if they are convinced that the psychological contract that binds them to their organization has been broken [60,61,62,63]. It emerges from this that organizational justice is measured through the perception of fairness [64] among employees vis-à-vis their organization in terms of the way decisions were made, as well as in relation to the distribution and even in the equity of the outcomes themselves [65]. It should be noted that at least two meta-analyses have discovered that procedural justice was negatively related to intentions to quit (e.g., [17,66]). Indeed, when procedural justice [19] is perceived as fair, employees are more engaged and their turnover intentions are lower [20,21,22]. This is shown in our conceptual framework (see Figure 1). Based on the aforementioned evidence, the first hypothesis of this research will take the following form:

**Hypothesis** **1** **(H1).** *The procedural justice will be negatively linked to turnover intent.*

Regarding social influence, Fischer [67] argues, “It generally indicates that one person’s action becomes a prescription for the direction of another person’s conduct”. We can therefore assume that social influence harbors everything that is likely to bring about, directly or indirectly, a mutation, in terms of behavior, inherent to the dominant pressures in a well-determined context. Moreover, on this subject, Moscovici [68] argues, “Individuals of many species learn to a large extent to know their environment through others” (p. 120). Moreover, Granovetter [69] considers that “agents” are “embedded” in networks of social relations, outside of which one cannot understand their behavior, i.e., the position of the agent in the network determines his behavior. The interest must therefore focus on the links, the relations between the agents and consequently, on their roles in the social network. Indeed, faced with unclear situations or tensions, the influence of the group is perceived as soothing or even sedative for some employees. Indeed, the group secures, provides support and removes the individual from the anxiety of failing.

For this reason, we assume that even if procedural justice exists and has been respected by top management, that employees, out of fear of negative judgments and under the influence of this mysterious power, may alter, without their knowledge, their opinions as to the procedures, which were, in all respects, followed. Thus, we believe that social influence has a positive effect on turnover intention while embodying a mediating role in the relationship that links procedural justice to turnover intention.

However, since social influence, as a mediating variable, has never been the subject of previous research, let alone in the relationship that links procedural justice to turnover, we are unable to present previous research that constitutes scientific evidence that can legitimize the hypotheses that we are going to make. This is what motivates us to move from explanatory research (The why) to exploratory research (The what) and “generate new ideas, conjectures, hypotheses”. Based on these arguments, we make the following hypotheses:

**Hypothesis** **2** **(H2).** *Social influence will be positively related to turnover intention*.

**Hypothesis** **3** **(H3).** *Procedural justice will be positively related to social influence*.

**Hypothesis** **4** **(H4).** *Social influence plays a mediating role between procedural justice and turnover intention*.

## 4. Methodology

### 4.1. Research Population and Sample

This research was conducted using a quantitative approach by means of a questionnaire, which was administered manually to 650 employees of all ranks and genders belonging to a banking institution, in this case “ABCDE Bank”, which extends over Tunisian territory, more precisely in Tunis, the capital. We were only able to receive 558 questionnaires that were usable, i.e., a return of 85.84%. Most of the respondents, 45.2% (of which 60.8% are women), were private, professional and corporate account managers, 25.7% (of which 10.2% are women) held director positions, such as managers, as well as back office managers and 18.6% (of which 8.5% are women) were managers. The rest of the sample, i.e., 10.5% (of which 45% are women), is made up of other managers (IT manager, organization, quality; IT specialist, quality manager). Regarding the 558 respondents, only 85.1% benefit from a permanent contract.

### 4.2. Measurement

First, a questionnaire was distributed to 150 employees of a few agencies scattered over the Tunisian territory to carry out the exploratory phase and consequently, the purification of the measurement scales. We were only able to collect 125 questionnaires being usable, i.e., a return of 83.33%. We opted for measurement scales in order to measure the expected variables. We would like to point out that a very specific approach was taken in the first version of the questionnaire. Indeed, the choice was made on measurement scales containing as few items as possible to motivate potential respondents to perform their tasks. All the items in the instruments were drawn from a pre-tested and well-defined scale. The procedural justice scale of Colquitt [66], turnover intention’s scale of Wayne et al. [70] and social influence scale of Ajzen [71] were adopted in the current study. All variables were measured using 5-point Likert-type scales ranging from 1 “completely disagree” to 5 “completely agree”.

## 5. Results

### 5.1. Confirmatory Factor Analysis

Confirmatory factor analysis (CFA) was used to verify the fit of the scale used to the data collected. The criteria for interpreting the results of a confirmatory factor analysis are numerous, often grouped into three categories. First, the absolute fit indices, which allow to assess the extent to which the theoretical model correctly reproduces the data collected. This is particularly the case for the parsimony index Chi2/ddl, the value of which must be less than 5 [72]; the SRMR, the value of which must be less than 0.05 and the RMSEA, the value which must be less than 0.08 and, if possible, 0.05 [73]. Then come the incremental indices, which are used to assess the improvement in the fit of the model being tested, compared to a more restrictive reference model. These are, more specifically, the NFI, the TLI and the CFI, the threshold value of which is 0.90 [74] and finally, the parsimony indices via the normalized x^2^.

The results of the first-order confirmatory factor analysis combining the dependent and independent variables of the study fit the data (Table 1). They display a Khi2 reported to its degree of freedom x^2^/ddl (4.724). A ratio is deemed satisfactory since it is less than 5. In addition, the RMSEA index is equal to 0.082, thus approaching zero, therefore, it shows us that the adjustment is satisfactory. The indices NFI = 0.992, TLI = 0.982 and CFI = 0.994 also authenticate the values accepted by the literature to offer a very good adjustment. The first-order results that arise from the exploratory factor analysis thus meet the recommended standards [75].

Two indicators are provided by the literature, in this case, the coefficient of symmetry (Skewness) and the coefficient of flattening (Kurtosis), able to compare the observed distribution with that of the normal law or Gaussian curve. The Skewness coefficient “shows whether the observations are distributed equitably around the mean (the coefficient is then zero) or if they are rather concentrated towards the lowest values (positive coefficient) or if they are rather concentrated towards the highest values. High (negative coefficient)” [68]. The Kurtosis coefficient compares “the shape of the observation distribution curve to that of the normal law: a positive coefficient indicates a higher concentration of observations while a negative coefficient indicates a flatter curve” [76]. Concerning our case, the coefficients of symmetry (Skewness) and kurtosis (Kurtosis) do not violate the assumption of normality [77] and reveal admissible values. We can conclude, in this respect, that all the distributions are fairly distributed and all the variables follow the normal law (Table 1).

In order to know whether the items of the variables, presumed to measure the same phenomenon, are correlated, we used convergent validity, which is, through the CR, must be strictly greater than 0.7 and the AVE, which must be strictly greater than 0.5. The results (Table 2) prove that convergent validity was verified for all variables [78]. To find out whether two theoretically distinct variables are also distinct in practice, we tested the discriminant validity. For this, it was necessary to check whether the square root of the AVE of each variable is strictly greater than the correlations it shares with the other variables. The results (Table 2) demonstrate that the discriminant validity was verified for the three variables.

To test the discriminant validity, we need the correlation matrix, the square roots of the AVEs and the Cronbach α specific to each variable (Table 2). The square roots of the AVEs are greater than the off-diagonal values, which represent the correlations between these constructs, thus confirming the discriminant validity of the factors as determined by Fornell and Larcker [79]. Additionally, the average extracted variance (AVE) scores for procedural justice (0.925), intention to turnover (0.875) and social influence (0.861) significantly outpace the maximum shared variances (MSV) that respectively represent the following values (0.291, 0.562, 0.562). Thus, as suggested by Hair et al. [80], discriminant validity is guaranteed. Moreover, the inter-correlation scores for each variable must not be greater than the values of the diagonal indicating the square roots of the AVEs specific to each factor (Table 2, in bold).

### 5.2. Structural Equation Modeling Results

Once the validity and reliability of the measures are ensured, we begin a structural equation modeling in order to verify the impact of organizational justice on turnover intentions specific to ABCDE Bank employees via social influence.

**Figure 2:**
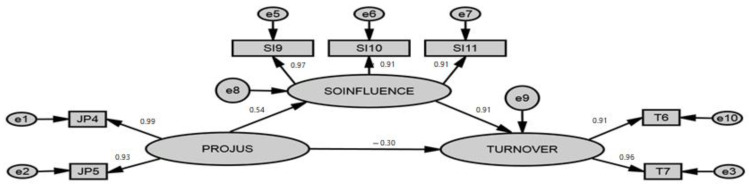
The structural model.

The results of the study fit the data (Table 3). They display a chi-square test or χ^2^ reported to its degree of freedom x^2^/ddl (3.155). A ratio is deemed satisfactory since it is less than 5. In addition, the RMSEA index is equal to 0.062, thus approaching zero, therefore, it shows us that the adjustment is satisfactory. The NFI = 0.994, TLI = 0.990 and CFI = 0.996 indices also authenticate the values accepted by the literature to offer a very good adjustment. The square root of the mean squared residuals RMR = 0.058 and the standardized RMR, SRMR = 0.062, turn out to be excellent since they are very close to zero. All the above hypotheses were verified and show significant relationships with *p* < 0.001 (Table 3, Figure 2). More specifically, procedural justice has a significant and negative effect on turnover intention (β = −0.30, *p* < 0.001), as well as a significant and positive effect on social influence (β = +0.54, *p* < 0.001), which has a significant and positive effect on turnover intention (β = +0.91, *p* < 0.001). Moreover, the robustness of the structural model is further legitimized by the significant coefficient of the value of (R^2^ = 0.563) (Table 3), which in our case, represents the proportion of turnover intention explained by procedural justice and the social influence in the regression model. Indeed, by using social influence and procedural justice, we can explain about 51% of turnover variance.

To test the mediating role of social influence in the relationship linking procedural justice to turnover intention, we used the approach of Baron and Kenny [81] which is an approach that represents a series of four successive tests. First, we must demonstrate that the link between procedural justice and turnover intention is significant in order to ensure the existence of an impact to be mediated. Indeed, procedural justice has a significant and negative effect on turnover intention (β = −0.30, *p* < 0.001). In the regression of turnover intention on procedural justice, the coefficient is significant (with a Student test equal to 5.595 ≥ 1.96; *p* = 0.05).

Second, we have to justify that procedural justice has a significant impact on the mediating variable, i.e., social influence, considered then as an exogenous variable in a regression analysis of social influence on procedural justice. Indeed, procedural justice has a significant and positive effect on social influence (β = +0.54, *p* < 0.001).

Third, we must confirm that the link between the mediating variable or even social influence and turnover intention is significant. Indeed, social influence has a significant and positive effect on turnover intention (β = +0.91, *p* < 0.001). Here, it is also a question of regressing turnover intention on both social influence and procedural justice. By controlling for the latter, the coefficient between social influence and turnover intention must remain significant as it is in our study (see Table 4, shaded lines).

Finally, we are led to verify the partial or complete nature of the social influence by examining the significance of the direct links between procedural justice and turnover intention. Moreover, the Sobel test gives us a value of Z = 11.80 > 1.96 with a *p*-value equal to 0 < 0.01. That said, we can conclude that social influence has a significant and partial mediation as a whole (see Table 3 and Table 4).

## 6. Discussion and Implications

Once again, the two meta-analyses, having found that procedural justice was negatively related to turnover intentions (e.g., [17,18]), were supported by our results. Our first hypothesis “Procedural justice will be negatively related to turnover intention” was confirmed (β = −0.30, *p* < 0.01) (see Table 3). Indeed, when procedures [19] are perceived as fair, employees are more committed and their turnover intentions become lower [20,21,22].

Nevertheless, contrary to a purely technical or Taylorist vision, organizations have an interest in taking into consideration, in addition to human resource management practices, certain organizational phenomena in the informal sector which, even in the presence of procedural justice, may even be of organizational justice, and still have negative consequences on the viability of the employee within his organization. Our hypotheses, having been confirmed, are proof of this. H2- Social influence will be positively related to turnover intention (β = +0.91, *p* < 0.01); and H4- Social influence plays a mediating role between procedural justice and turnover intention, with a Sobel test, (Z = 2.26 > 1.96, *p*-value = 0 < 0.01) (See Table 3 and Table 4). By this, we insinuate that social influence is an organizational phenomenon that is all the more dangerous as it is difficult to control. Indeed, the act of influencing does not necessarily mean the possession of characteristics of power or a superior social rank. Moreover, Boudon and Lazarsfeld [82] define the influence structure as “the distribution of decision-making poles in an organization; it does not necessarily correspond to the formal authority structure. The simplest determination of the poles of influence consists in observing who, in the event of a conflict, obtains the advantage” (p. 83).

In the case of ABCDE Bank, we are witnessing a majority-type influence called in social psychology conformism. This explains the result, certainly unreasonable, but quite explainable to which we have reached. Indeed, the last hypothesis stipulating “procedural justice has a significant and positive effect on social influence” (β = +0.54, *p* < 0.001) has been confirmed. Here, ABCDE Bank is confronted with a phenomenon that appears when an individual, forced to prescribe himself to the hegemony of the norms of the majority group, proceeds to a transformation of his thoughts and his behavior, even if a priori the procedures are fair and quite appropriate. Moscovici [83] supports the idea that “conformity takes place when an individual modifies his behavior or attitude in order to bring it into better harmony with the behavior or attitude of a group” (p. 26). This behavioral change, faced with an existing procedural justice within ABCDE Bank, must comply with the integration criteria often prescribed by the majority vis-à-vis those who are motivated to integrate it. Fischer [67] denotes three stages in the compliance process. The first alludes to a situation of ambivalence felt by the individual, concretized by a cognitive antagonism opposing his previous ways of doing and thinking to the pressures to which he is summoned to submit. The second level has the main interest of showing the weight and impact of social influence on the individual, which is an obviousness illustrated by his accommodation compared to all that is recommended to him. The last level states the result of the transformation justified by a certain self-realization felt by the individual and this is after his alignment with the new behaviors and attitudes; but again and, above all, by a voluntary or unconscious renunciation on his part of certain behaviors adopted and undertaken previously.

Here, the social comparison of Festinger [84,85] may be the source of social influence. First, because no one can already contradict that this phenomenon is spreading within groups. Second, the work of Deutsch and Gérard [86], in the field of informative influence, supports the idea that when individuals are skeptical about the validity of their opinions, they experience a certain anxiety in the sense of Schachter [87]. Indeed, they will tend to conform to the group to which they belong, for two reasons. On the one hand, because they assume that the judgment of the group is more accurate than their own, hence the phenomenon of social comparison by Festinger [84,85], and on the other hand, to reduce the weight of the uncertainty hanging over them. In general, this informative influence implies, according to Moscovici [83], on the part of the influenced subject, both “public submission and private acceptance” (p. 31). Rightly, Moscovici [88] advances: “It happens to each of us to change opinions and behaviors by obscure ways which escape reason. In a subterranean way or almost, one would be tempted to say”.

The empirical results of our research that we have been able to reach can be of great benefit to policy makers and senior management in particular, especially in the banking industry in both national and international organizations. Our research showed that social influence has a significant impact on the relationship between procedural justice and turnover intention. Hence, the top management should be aware of this influence and undertake proper human resource management practices to deal with such a phenomenon as it is very likely to harm the performance of the organization, which will increase its chances of retaining its human asset.

## 7. Conclusions

We believe, in all modesty, that the main theoretical contribution of this research is that it contributes to enriching Folger’s Theory of Cognitive References [23]. This theory focuses, among other things, on the procedures that serve as vectors for decision-making. The CRT further reveals that favorable perceptions of procedural justice offset unfavorable perceptions of distributive justice. Poor procedures and vague and confusing explanations adopted by top management will make the distribution decision feel arbitrary, since in their heads, employees invent allocation or explanation scenarios that are better than those actually experienced. This psychological discomfort will materialize in a certain animosity or even in aversion felt by employees towards top management. As paradoxically as it may seem, a real distributive injustice, carried out by top management with regard to its employees, will be completely concealed if it is conveyed by a few fair procedures and sufficient or even reassuring explanations. Conversely, the absence of a fair procedure for employees encourages them to value or even amplify unfair distribution by attributing disproportionate attention to it, even if it is a priori a tiny, almost negligible injustice.

## 8. Limitation and Future Research Directions

This research was undertaken on a homogeneous group of workers in a single case study of ABCDE Bank in the capital city of Tunis, Tunisia. Hence, the results cannot be simply generalized to other professions or other countries’ contexts without further testing these results. Future research opportunities could replicate the research model in other countries’ contexts, especially those with different cultural influences. Other opportunities could include examining the meditating role of social influence between the three constructs of justice and turnover intention. Additionally, impact of leadership styles on social influences and turnover intentions would also be an interesting future topic of research study.

## Figures and Tables

**Table 1:** Descriptive statistics (developed by authors based on previous literature).

Abbr.	Item	Min	Max	M	SD	Skewness	Kurtosis
Procedural Justice							
JP4	I have the opportunity to express my point of view and my feelings during decision-making procedures.	1	5	3.03	1.583	−0.018	−1.593
JP5	The procedures applied allow me to have influence on the results of the decisions that concern me.	1	5	2.89	1.550	0.035	−1.578
Turnover Intention							
T6	I often think about quitting my job.	1	5	3.53	1.437	−0.640	−1.017
T7	It would not be worth much to make me quit my job.	1	5	3.51	1.532	−0.647	−1.149
Social Influence							
SI9	Usually, I tend to take into account the opinion of my acquaintances.	1	5	3.92	1.344	−1.203	0.215
SI10	Usually, I tend to consider my friends’ opinions.	1	5	3.83	1.281	−1.101	0.123
SI11	Usually, I tend to consider my family’s opinion.	1	5	3.60	1.457	−0.840	−0.693

Model fit: (χ^2^ (9, N = 525) = 24,030, *p* < 0.001, normed χ^2^ = 2.67, RMSEA = 0.082, SRMR = 0.0222, CFI = 0.994, TLI = 0.982, NFI = 0.992, PCFI = 0.762 and PNFI = 0.688). Note: Min = minimum, Max = maximum, M = mean, SD = standard deviation.

**Table 2:** Convergent and discriminant validity (developed by authors).

Factors and Items	Standardized Loading	CR	AVE	MSV	ASV	1	2	3
1—Procedural Justice Colquitt [66] (α = 0.960)		0.923	0.857	0.291	0.163	0.925		
- I have the opportunity to express my point of view and my feelings during decision-making procedures.	0.990							
- The procedures applied allow me to have influence on the results of the decisions that concern me.	0.932							
2—Turnover Intention Wayne et al. [70] (α = 0.931)		0.867	0.766	0.562	0.426	0.153 **	0.875	
- I often think about quitting my job.	0.907							
- It would not be worth much to make me quit my job.	0.962							
3—Social Influence Ajzen, [71] (α = 0.952)		0.896	0.742	0.562	0.299	0.499 **	0.713 **	0.861
- Usually, I tend to take into account the opinion of my acquaintances.	0.967							
- Usually, I tend to consider my friends’ opinions.	0.910							
- Usually, I tend to consider my family’s opinion.	0.905							

AVE = Average Variance Extracted, MSV = Maximum Shared Value, ASV = Average Shared Value. ** = *p* < 0.01

**Table 3:** Result of the structural model (developed by authors).

Result of the Structural Model	β	C-R T-Value	R^2^	Hyp. Results
H1—Procedural justice → Turnover intention	−0.30 ***	26.161		Supported
H2—Social influence → Turnover intention	0.91 ***	23.949		Supported
H3—Procedural justice → Social influence	0.54 ***	13.575		Supported
H4—Procedural justice → Social influence → Turnover intention	*p*-1: β = 0.54 *** *p*-2: β = 0.91 ***	*p*-1: t-value = 13.575 *p*-2: t-value = 23.949		Supported
Turnover intention			0.563	

*** = *p* < 0.001.

**Table 4:** Type of mediation (developed by authors).

User-Defined Estimands: (Group Number 1—Default Model)					Mediation
Parameter	Estimate	Lower	Upper	*p*	
H4—Procedural justice → Social influence → Turnover intention	0.439	0.369	0.499	0.006	0.006 < 0.05 Partial Mediation

## Data Availability

Data is available upon request from researchers who meet the eligibility criteria. Kindly contact the first author privately through e-mail.

## References

[1] Renaud S., Tremblay F.A., Morin L. (2014). L’impact de la Justice Organisationnelle sur la Fidélisation: Étude Longitudinale auprès de Travailleurs du Secteur des TIC au Canada. Quest. Manag..

[2] Sobaih A.E.E., Hasanein A.M., Aliedan M.M., Abdallah H.S. (2020). The impact of transactional and transformational leadership on employee intention to stay in deluxe hotels: Mediating role of organisational commitment. Tour. Hosp. Res..

[3] Aryani R., Widodo W., Chandrawaty C. (2021). How Adversity Quotient and Organizational Justice Reduce Turnover Intention Empirical Evidence from Indonesia. J. Asian Financ. Econ. Bus..

[4] Putri G.C., Hasanati N. (2022). Individual and Situational Factors: Literature Review Predictors of Turnover Intention. Am. Res. J. Humanit. Soc. Sci..

[5] Caillier J.G. (2021). The impact of workplace aggression on employee satisfaction with job stress, meaningfulness of work, and turnover intentions. Public Pers. Manag..

[6] Demircioglu M.A., Berman E. (2019). Effects of the Innovation Climate on Turnover Intention in the Australian Public Service. Am. Rev. Public Adm..

[7] Thoresen C.J., Kaplan S.A., Barsky A.P., Warren C.R., De Chermont K. (2003). The Affective Underpinnings of Job Perceptions and Attitudes: A Meta-Analytic Review and Integration. Psychol. Bull..

[8] Esteves T., Lopes M.P. (2020). Crafting a Calling: The Mediating Role of Calling between Challenging Job Demands and Turnover Intention. J. Career Dev..

[9] Jacobs E., Roodt G. (2007). The Development of a Knowledge Sharing Construct to Predict Turnover Intentions. Aslib Proc. New Inf. Perspect..

[10] Waldman J.D., Kelly F., Arora S., Smith H.L. (2010). The Shocking Cost of Turnover in Health Care. Health Care Manag. Rev..

[11] Mitrovska S. (2016). Calculating the Cost for Employee Turnover in the IT Industry. J. Hum. Resour. Manag..

[12] Gharbi H., Aliane N., Sobaih A.E.E. (2022). I Trust You: Does This Matter in the Relationship between Sexual Harassment, Continuous Commitment and Intention to Leave among Young Female Healthcare Professionals?. Int. J. Environ. Res. Public Health.

[13] Alyahya M.A., Elshaer I.A., Sobaih A.E.E. (2021). The Impact of Job Insecurity and Distributive Injustice Post COVID-19 on Social Loafing Behavior among Hotel Workers: Mediating Role of Turnover Intention. Int. J. Environ. Res. Public Health.

[14] Elshaer I.A., Azazz A.M. (2021). Amid the COVID-19 Pandemic, Unethical Behavior in the Name of the Company: The Role of Job Insecurity, Job Embeddedness, and Turnover Intention. Int. J. Environ. Res. Public Health.

[15] Sobaih A.E.E., Ibrahim Y., Gabry G. (2019). Unlocking the black box: Psychological contract fulfillment as a mediator between HRM practices and job performance. Tour. Manag. Perspect..

[16] Griffeth R.W., Hom P.W., Gaertner S. (2000). A Meta-Analysis of Antecedents and Correlates of Employee Turnover: Updated Moderator Tests and Research Implications for the Next Millennium. J. Manag..

[17] Cohen-Carash Y., Spector P.E. (2001). The role of justice in organizations: A meta-analysis. Organ. Behav. Hum. Decis. Process..

[18] Colquitt J.A., Conlon D.E., Wesson M.J., Porter C.O., Ng K.Y. (2001). Justice at the Millennium: A Meta-Analytic Review of 25 Years of Organizational Justice Research. J. Appl. Psychol..

[19] Brockner J., Wiesenfeld B.M., Greenberg J., Colquitt J.A. (2005). How, When, and Why Does Outcome Favorability Interact with Procedural Justice?. Handbook of Organizational Justice.

[20] Gim G.C., Desa N.M. (2014). The Impact of Distributive Justice, Procedural Justice, and Affective Commitment on Turnover Intention among Public and Private Sector Employees in Malaysia. Int. J. Soc. Sci. Humanit..

[21] Mengstie M.M. (2020). Perceived Organizational Justice and Turnover Intention among Hospital Healthcare Workers. BMC Psychol..

[22] Chong A., Hussain I.A., Ahmad N., Singh J.S.K. (2021). Organisation Justice Towards’ Employees Voluntary Turnover: A Perspective of SMEs in Malaysia. Int. J. Hum. Resour. Stud..

[23] Folger R., Olson J.M., Herman C.P., Zanna M.P. (1986). A Referent Cognitions Theory of Relative Deprivation. Social Comparison and Relative Deprivation: The Ontario Symposium.

[24] Bellenger L. (2004). Réussissez Toutes Vos Négociations.

[25] Williams K.D. (2007). Ostracism. Annu. Rev. Psychol..

[26] Maslow A.H. (1943). A Theory of Human Motivation. Psychol. Rev..

[27] Matthews R.A., Ritter K.-J. (2019). Applying Adaptation Theory to Understand Experienced Incivility Processes: Testing the Repeated Exposure Hypothesis. J. Occup. Health Psychol..

[28] Namin B.H., Øgaard T., Røislien J. (2022). Workplace Incivility and Turnover Intention in Organizations: A Meta-Analytic Review. Int. J. Environ. Res. Public Health.

[29] Saleh T.A., Mehmood W., Khan J., Jan F.U. (2022). The Impact of Ethical Leadership on Employees Turnover Intention: An Empirical Study of the Banking Sector in Malaysia. J. Asian Financ. Econ. Bus..

[30] Xiong R., Wen Y. (2020). Employees’ Turnover Intention and Behavioral Outcomes: The Role of Work Engagement. Soc. Behav. Pers..

[31] Lin C.-Y., Huang C.-K. (2020). Employee Turnover Intentions and Job Performance from a Planned Change: The Effects of an Organizational Learning Culture and Job Satisfaction. Int. J. Manpow..

[32] Mai K.E., Ellis A.P.J., Christian J.S., Porter C.O. (2016). Examining the Effects of Turnover Intentions on Organizational Citizenship Behaviors and Deviance Behaviors: A Psychological Contract Approach. J. Appl. Soc. Psychol..

[33] Shkoler O., Tziner A. (2017). The Mediating and Moderating Role of Burnout and Emotional Intelligence in the Relationship between Organizational Justice and Work Misbehavior. Rev. Psicol. Trabajo Organ..

[34] Gharbi H., Sobaih A.E. (2022). Developing and Validating a Scale Measuring Sexual Harassment in Higher Education. Dirasat-Hum. Soc. Sci..

[35] Fishbein M., Ajzen I. (1997). Belief, Attitude, Intention and Behavior: An Introduction to Theory and Research.

[36] Maertz C.P., Keith M.G., Raghuram S., Porter C.M., Dalton G.L. (2022). Advancing Theory and Practice on Managing Dysfunctional Turnover: Developing an Improved Measure of Turnover Reasons. Group Organ. Manag..

[37] Ma Y., Chen F., Xing D., Meng Q., Zhang Y. (2022). Study on the Associated Factors of Turnover Intention among Emergency Nurses in China and the Relationship between Major Factors. Int. Emerg. Nurs..

[38] Amponsah-Tawiah K., Annor F., Arthur B.G. (2016). Linking Commuting Stress to Job Satisfaction and Turnover Intention: The Mediating Role of Burnout. J. Workplace Behav. Health.

[39] Wen B., Zhou X., Hu Y., Zhang X. (2020). Role Stress and Turnover Intention of Front-Line Hotel Employees: The Roles of Burnout and Service Climate. Front. Psychol..

[40] Effendi M., Nimran U., Utami H.N., Afrianty T.W. (2021). Effects of Psychological Capital and Gratitude on Employees Intention to Leave: The Role of Job Satisfaction. J. Asian Financ. Econ. Bus..

[41] Hirst G., Van Dick R., Van Knippenberg D. (2009). A Social Identity Perspective on Leadership and Employee Creativity. J. Organ. Behav..

[42] Kim D., Vandenberghe C. (2020). Ethical Leadership and Team Ethical Voice and Citizenship Behavior in the Military: The Roles of Team Moral Efficacy and Ethical Climate. Group Organ. Manag..

[43] Al Muharraq E.H., Baker O.G., Alallah S.M. (2022). The Prevalence and the Relationship of Workplace Bullying and Nurses Turnover Intentions: A Cross Sectional Study. SAGE Open Nurs..

[44] Osman I., Noordin F. (2015). The Impact of Organisational Justice, Organisational Trust and Teamwork on Talent Retention: Mediating Role of Organisational Citizenship Behaviour. Adv. Sci. Lett..

[45] Hom P.W., Lee T.W., Shaw J.D., Hausknecht J.P. (2017). One Hundred Years of Employee Turnover Theory and Research. J. Appl. Psychol..

[46] Deutsch M. (1985). Equity, Equality, and Need: What Determines Which Value Will Be Used as the Basis of Distributive Justice?. J. Soc. Issues.

[47] Greenberg J. (1990). Looking Fair vs. Being Fair: Managing Impressions of Organizational Justice. Res. Organ. Behav..

[48] Tyler T.R., Lind E.A. (1992). A Relational Model of Authority in Groups. Adv. Exp. Soc. Psychol..

[49] Adams J.S. (1963). Toward an Understanding of Inequity. J. Abnorm. Psychol..

[50] Adams J.S. (1965). Inequity in Social Exchange. Exp. Soc. Psychol..

[51] Thibaut J., Walker L. (1978). A theory of procedure. Calif. L. Rev..

[52] Bies R.J., Moag J.F., Lewicki R.J., Sheppard B.H., Bazerman M. (1986). Interactional justice: Communication criteria of fairness. Research on Negotiations in Organization.

[53] Tyler T.R., Bies R.J., Carroll J.S. (1990). Beyond Formal Procedures: The Interpersonal Context of Procedural Justice. Applied Social Psychology in Organizational Settings.

[54] Cialdini R. (1984). Influence.

[55] Tarde G. (1903). The Laws of Imitation.

[56] Gouldner A.W. (1960). The Norm of Reciprocity: A Preliminary Statement. Am. Sociol. Rev..

[57] Homans G.C. (1961). Social Behavior: Its Elementary Forms.

[58] Blau P. (1964). Exchange and Power in Social Life.

[59] Blau P. (1994). Structural Contexts of Opportunities.

[60] Argyris C. (1960). Understanding Organizational Behavior.

[61] Levinson H., Price C.R., Munden K.J., Solley C.M. (1962). Men, Man—Agement and Mental Health.

[62] Schein E.H. (1965). Organizational Psychology.

[63] Rousseau D.M. (1989). Psychological and Implied Contracts in Organizations. Empl. Responsib. Rights J..

[64] Schultz D., Schultz S.E. (2016). Psychology and Work Today.

[65] Asadullah M.A., Akram A., Imran H., Arain G.A. (2017). When and Which Employees Feel Obliged: A Personality Perspective of How Organizational Identification Develops. Rev. Psicol. Trabajo Organ..

[66] Colquitt J.A. (2001). On the Dimensionality of Organizational Justice: A Construct Validation of a Measure. J. Appl. Psychol..

[67] Fischer G., Fischer G. (2020). L’influence Sociale. Les Concepts Fondamentaux de la Psychologie Sociale.

[68] Moscovici S. (1994). Psychologie Sociale des Relations à Autrui.

[69] Granovetter M. (1985). Economic Action and Social Structure: The Problem of Embeddeeness. Am. J. Sociol..

[70] Pedhazur E.J., Pedhazur Schmelkin L. (1991). Measurement, Design, and Analysis: An Integrated Approach.

[71] Wayne S.J., Shore L.M., Liden R.C. (1997). Perceived organizational support and leader–member exchange: A social exchange perspective. Acad. Manag. J..

[72] Ajzen I. (1991). The Theory of Planned Behavior. Organ. Behav. Hum. Decis. Process..

[73] Roussel P., Roussel P., Wacheux F. (2005). Méthodes de Développement d’Échelles pour Questionnaires d’Enquête. Management des Ressources Humaines: Méthodes de Recherche en Sciences Humaines et Sociales.

[74] Bentler P.M., Bonett D.G. (1980). Significance Tests and Goodness-Of-Fit in the Analysis of Covariance Structures. Psychol. Bull..

[75] Roussel P., Durrieu F., Campoy E., El Akremi A. (2002). Méthodes d’Équations Structurelles: Recherche et Applications en Gestion.

[76] Evrard Y., Pras B., Roux E. (2000). Market: Etudes et Recherches en Marketing.

[77] Kline R.B. (2015). Principles and Practice of Structural Equation Modeling.

[78] Joreskog K.G., Cattell R.B., Nessclroade J.R. (1988). Analysis of Covariance Structures. The Handbook of Multivariate Experimental Psychology.

[79] Fornell C., Larcker D. (1981). Evaluating structural equation models with unobservable variables and measurement error. J. Mark. Res..

[80] Hair J., Anderson R., Tatham R., Black W. (2014). Multivariate Data Analysis.

[81] Baron R.M., Kenny D.A. (1986). The Moderator–Mediator Variable Distinction in Social Psychological Research: Conceptual, Strategic, and Statistical Considerations. Pers. Soc. Psychol..

[82] Boudon R., Lazarsfeld P. (1965). Le Vocabulaire des Sciences Sociales.

[83] Moscovici S. (1998). Psychologie Sociale.

[84] Festinger L. (1950). Informal Social Communication. Psychol. Rev..

[85] Festinger L. (1954). A Theory of Social Comparison Processes. Hum. Relat..

[86] Deutsch M., Gérard H.B., Faucheux C. (1955). Etude Des Influences Normatives et Informationnelles sur le Jugement Individuel. Psychologie Sociale Théorique et Expérimentale, Paris, Maloine (1971). Cité par Fischer, G-N. (1987). Les Concepts Fondamentaux de la Psychologie Sociale.

[87] Schachter S. (1959). The Psychology of Affiliation.

[88] Moscovici S., Berkowitz L. (1980). Toward a Theory of Conversion Behavior. Advances in Experimental Social Psychology.

